# Supplementary material for: Limitations of calculating theoretical solutions for closed BCMP queueing networks and verification of alternative theoretical values by parallel simulation
Source: PLoS One. 2024 Dec 17;19(12):e0311533. doi: 10.1371/journal.pone.0311533 (PMC11651580; doi:10.1371/journal.pone.0311533)
Supplement: Attachment — Submitted filename: response_retter_202407.docx [file pone.0311533.s001.docx]

Dear Dr. Sathyaprakash,

We would like to thank the reviewers for their valuable comments and suggestions. We have carefully considered each comment and have made the necessary revisions to our manuscript. Below, we provide detailed responses to each comment.

*Reviewer #1: This paper is applied a closed BCMP queueing network to a real-world model and examining the limitations of the theoretical solution, The paper needs some improvements as listed below.*

1. *Comment: The abstract needs to be a little bit more precise and numerically conclusive.*

Response: Thank you for your valuable comment on the abstract. We have revised the abstract to include more precise and numerically conclusive information, as follows:

"This study applied a closed BCMP queueing network to a real-world model, examining the limitations of the theoretical solution and the possibility of replacing theoretical values with those from parallel simulation. Parallel computing was applied to mean value analysis (MVA). We first obtained computational and theoretical values by varying the number of nodes from 33 to 300 and customers from 250 to 1500 in a system with three customer classes. The computation time increased proportionally with the number of nodes but exponentially with the number of customers, reaching 146,798.86 seconds for 33 nodes, 3 customer classes, and 1500 customers. We then considered a system with more customer classes; due to the greater computational burden, we proposed addressing this problem with simulation. By using a large-scale computing environment (a supercomputer), it was possible to obtain the theoretical solutions for up to three customer classes and verify the simulation accuracy. The parallel simulations' performance-evaluation indices, such as the average number of people in the system, converged to the theoretical values within an acceptable error range after 100,000 simulation hours for systems with four or more customer classes. These results demonstrate that the proposed parallel simulation approach can serve as an accurate and computationally efficient alternative to theoretical solutions for large-scale closed BCMP queueing networks."

The revised abstract now includes specific numerical details, such as the range of nodes and customers considered, the computation time for a particular case, and the acceptable error range and simulation hours required for systems with more customer classes. These additions make the abstract more precise and provide a clearer picture of the study's findings.

2. *Comment: Introduction can be more impressive in terms of Identifying the Research Problem or Gap, State the Research Objectives, Outline the Scope and Methodology, Highlight the Significance of the Study and Provide an Overview of the Paper Structure, so, author should rewrite the introduction.*

Response: We appreciate your suggestion to make the introduction more impressive by better addressing the key components of a well-structured introduction. We have now rewritten the introduction to incorporate the following elements:

1. Identifying the research problem or gap: We have highlighted the computational complexity involved in applying queueing theory to large-scale systems and the limitations of existing methods when dealing with four or more customer classes.

2. Stating the research objectives: We have clearly stated our aims to investigate the computational limits of exact methods for solving large-scale closed BCMP queueing networks and to propose an alternative parallel simulation approach for cases where exact solutions become intractable.

3. Outlining the scope and methodology: We have mentioned the use of mean value analysis (MVA) for exact solutions and the development of a parallel simulation approach as an alternative method. The scope of our study includes closed BCMP queueing networks with varying numbers of nodes, customer classes, and people in the network.

4. Highlighting the significance of the study: We have emphasized the importance of queue management in the context of the COVID-19 pandemic and the role of queueing theory in redesigning public spaces and facilities. We have also pointed out the potential of our proposed parallel simulation approach in enabling the analysis of large-scale queueing networks that were previously intractable.

5. Providing an overview of the paper structure: We have added a paragraph at the end of the introduction that outlines the structure of the paper, giving the reader a clear idea of what to expect in each section.

By incorporating these elements, the revised introduction now provides a more impressive and comprehensive overview of our research, effectively communicating the motivation, objectives, methodology, and significance of our work.

3. *Comment: Related work should be biased on latest work and they should cite the latest research papers too.*

Response: Thank you for your feedback on the Related Work section and Introduction. We have made substantial improvements to address your concerns:

1. Incorporation of recent research: We have added information on recent studies focusing on large-scale closed queueing networks, machine learning integration, and approximation methods (Patel and Bhathawala [19], Li and Fang [20], Osakwe et al. [21], Sato and Yokota [22], Chen and Yuan [23]).
2. COVID-19 related research: We have emphasized the relevance to current social conditions by including studies on social distancing during the pandemic (Chu et al. [10]) and its impact on transportation systems (Jiang et al. [11]).
3. Advancements in computational methods: We have added recent research on computational methods for queueing networks, including parallel computing (Mesheryakov et al. [13]) and active queue management (Awan et al. [12]).
4. Analysis of closed queueing networks: We have strengthened the theoretical foundation for performance analysis of closed queueing networks by incorporating the works of Lagershausen [15], Rege [16], and Bertsimas et al. [17].
5. Expanded scope: The revised sections now cover a broader range of topics, including non-exponential service times, parallel simulation approaches, deep reinforcement learning applications, and approximate analysis techniques for load-dependent service times.
6. Relevance to current trends: We have highlighted the growing integration of queueing theory with machine learning and its application to modern systems such as smart factories.
7. Consistency in referencing: We have updated the reference numbers throughout the sections to maintain consistency with the newly added literature.

These changes provide a more comprehensive and up-to-date overview of the field, better contextualizing our research within current trends and developments in closed queueing network analysis and applications. Additionally, they clearly demonstrate the relevance of our work to contemporary challenges, such as the impact of the COVID-19 pandemic and the latest computational methods.

(add to Introduction)

10.	D.K. Chu, E.A. Akl, S. Duda, K. Solo, S. Yaacoub, H.J. Schünemann, et al., Physical distancing, face masks, and eye protection to prevent person-to-person transmission of SARS-CoV-2 and COVID-19: a systematic review and meta-analysis, The Lancet 395 (2020) 1973–1987.

11.	R. Jiang, S. Shen, Y. Zhang, Analyzing the impact of COVID-19 on transportation systems using queueing theory, Transportation Research Part A: Policy and Practice 145 (2021) 244-262.

12.	Awan, I., Ahmad, B., & Ahmad, S. (2007). Performance analysis of networks of queues under active queue management scheme. Simulation Modelling Practice and Theory, 15(4), 416-425.

13.	Mesheryakov, R., Moiseev, A., Demin, A., Dorofeev, V., & Sorokin, V. (2016). Using Parallel Computing in Queueing Network Simulation. In Key Engineering Materials (Vol. 685, pp. 943–947). Trans Tech Publications, Ltd. https://doi.org/10.4028/www.scientific.net/kem.685.943

15.	S. Lagershausen, Performance analysis of closed queueing networks, Lecture Notes in Economics and Mathematical Systems, Springer, 2013.

16.	K.M. Rege, Multiclass queueing models for performance analysis of computer systems, Sadhana 15 (1990) 355-363.

17.	D. Bertsimas, D. Gamarnik, J.N. Tsitsiklis, Performance analysis of multiclass queueing networks, SIGMETRICS Performance Evaluation Review 27 (1999) 11-14.

(add to related work)

19.	R. Patel, P.H. Bhathawala, Performance analysis of closed queueing networks with multiple classes and non-exponential service times, Journal of Industrial and Management Optimization 17 (2021) 1825–1842.

20.	Z. Li, W. Fang, Parallel simulation of large-scale closed queueing networks with multi-class customers, in: 2020 IEEE 22nd International Conference on High Performance Computing and Communications, 2020, pp. 822–829.

21.	I.C. Osakwe, A. Aneiba, S. Misra, Performance optimization of queuing systems using deep reinforcement learning, in: Machine Learning for Predictive Analysis, Springer, Cham, 2021, pp. 25-42.

22.	K. Sato, Y. Yokota, Modeling and performance evaluation of a closed queueing network for a smart factory, in: 2020 IEEE International Conference on Industrial Engineering and Engineering Management (IEEM), 2020, pp. 1165-1169.

23.	X. Chen, D. Yuan, Approximate analysis of closed queueing networks with load-dependent service times, Performance Evaluation 148 (2021) 102180.

4. *Comment: In this paper, the author provided a theoretical and practical comparison of the results, whether the author should compare their results with the other (same area) paper’s results because in research papers we are working to improve the already done work and result.*

Response: We appreciate the reviewers' comments on the importance of comparing our results with those of other studies in the same area. In this study, we have focused on applying the closed BCMP queueing network to a large-scale real-world model, which distinguishes our work from previous studies.

As shown in Table 1, our study considers a significantly larger number of nodes, customer classes, and people in the network compared to earlier research. This demonstrates the novelty and importance of our work in addressing the challenges of analyzing large-scale systems. We have highlighted this point in the revised Section 1.2 "Contributions of this study" as follows:

"i. For closed BCMP, we used MVA, an exact method for obtaining theoretical solutions, to show the limits computable in a modern computing environment. The performance-evaluation index of the closed BCMP could be obtained adequately for large-scale networks, especially when the number of customer classes was three or less. The scale of the systems considered in this study, in terms of the number of nodes, customer classes, and people in the network, is significantly larger than those in previous studies, as shown in Table 1. This demonstrates the novelty and importance of our work in tackling the challenges of large-scale system analysis."

Moreover, we have successfully parallelized the Mean Value Analysis (MVA) algorithm, enabling us to calculate theoretical values for large-scale systems with up to three customer classes. This contribution is significant, as it allows for the analysis of large-scale systems that were previously difficult to handle using conventional methods.

Regarding the simulations, we first verified the accuracy of our approach for large-scale systems with three or fewer customer classes. We then applied simulations to systems with a larger number of customer classes, demonstrating that our proposed method can produce results even for these more complex scenarios. This highlights the effectiveness and broad applicability of our approach. We have emphasized this contribution in the revised Section 1.2:

"iii. Using simulations with verified accuracy, we calculated performance metrics for closed BCMP with four or more customer classes. This simulation-based approach serves as an efficient alternative to exact methods for analyzing large-scale closed BCMP queueing networks that were previously intractable."

While we acknowledge the importance of comparing our results with those of other studies, the unique scale and focus of our work make direct comparisons challenging. However, we believe that our study contributes to the advancement of the field by addressing the limitations of existing methods and providing a novel approach for analyzing large-scale closed BCMP queueing networks.

5. *Comment: Overall, an English translation is grammatically sound but the order of placement of topics often goes wrong and reading patterns get out of hand.*

Response: Thank you for your suggestion to improve the structure of the document and the order of topics. To address this comment, we have made the following changes:

1. We have moved the section "2.5 Massively Parallel Computing Environment for MVA" to the beginning of Chapter 3, renumbering it as "3.1 Massively Parallel Computing Environment for MVA."
2. We have added an overview of the chapter's structure directly under the title "3. Results and Discussion," which outlines the flow of the topics and how they are interconnected. The added text is as follows:

"In this chapter, we present and discuss the results of our study on the limitations of calculating theoretical values for closed BCMP queueing networks and the effectiveness of parallel simulation as an alternative method. We begin by describing the massively parallel computing environment used for the mean value analysis (MVA) in Section 3.1. In Section 3.2, we examine the factors affecting the calculation of the theoretical solution for a closed BCMP queueing network, followed by a consideration of the number of parallels for MVA parallel computation in Section 3.3.

Next, we propose the use of parallel simulation to calculate performance metrics for closed BCMP when the number of customer classes is large, making MVA computationally challenging. We verify the accuracy of parallel simulations in Section 3.4.1 and present the results of using parallel simulation as an alternative to theoretical values in Section 3.4.2.

Finally, in Section 3.5, we discuss the limitations of calculating theoretical values for closed BCMP and the effects of parallel simulation, highlighting the contributions and implications of our study."

These modifications help to clarify the structure of the document and provide a logical order for the topics, making it easier for readers to follow the flow of the research and understand the connections between the different sections.

6. *Comment: Since the author did not cite any sources for equations No. 1 through No. 9, it is unclear how real and true they are.*

Response: Thank you for your comment regarding the lack of citations for equations (1) through (9), which led to uncertainty about their reliability. To address this concern, we have made the following modifications directly in the relevant sections of the manuscript:

In Section 2.1, we clarified that equations (1) to (5) describe the fundamental properties of the closed BCMP queueing network model and cited the original BCMP paper by Baskett et al. [3].

In Section 2.2, we specified that equations (6) to (8) represent the basic steps of the mean value analysis (MVA) method for closed BCMP queueing networks and indicated that they are based on the description provided in Bolch et al. [4].

In Section 2.3, we explained that equation (9) is an original set definition introduced in this study to calculate the overlap combination for each iteration j in the proposed parallel computation algorithm for MVA.

In Section 2.4, we stated that equation (10) is an original formula for evaluating the error precision of the parallel simulation approach proposed in this study.

These modifications help to clarify the origin and reliability of the equations and address the reviewer's concerns. In the future, we will ensure that appropriate citations are provided, and original contributions are clearly indicated to maintain the credibility of the equations used in our work.

7. *Comment: Working steps (algorithm) is missing.*

Response: We appreciate your comment regarding the missing working steps. We have addressed this by clearly outlining the algorithms for both the parallel MVA computation and the event-driven parallel simulation in Sections 2.3 and 2.4, respectively. These algorithms now provide a step-by-step description of the proposed methods, enhancing the clarity and reproducibility of our work.

8. *Comment: Include Flow chart and symbol table with this work.*

Response: Thank you for suggesting the inclusion of flowcharts and a symbol table. We have incorporated two flowcharts to visualize our proposed approaches:

1. Fig. 1 in Section 2.3 illustrates the Parallel Computation Algorithm for Mean Value Analysis (MVA) using Message Passing Interface (MPI).
2. Fig. 2 in Section 2.4 depicts the Event-Driven Parallel Simulation Algorithm for BCMP Queueing Networks.

These flowcharts provide a clear visual representation of our methods, enhancing the understanding of the proposed algorithms. Additionally, we have added detailed explanations of these flowcharts in the text to further clarify the process.

Regarding the symbol table, we have included definitions of all symbols used throughout the paper in the relevant sections. However, if you feel a comprehensive symbol table would be beneficial, we can certainly add one in a future revision.

9. *Comment: Overall, an English translation is grammatically sound but the order of placement of topics often goes wrong and reading patterns get out of hand.*

Response: We appreciate your feedback on the order of topics and the overall readability of the manuscript. As mentioned in our response to your previous comment (Comment 5), we have taken the following steps to address these concerns:

1. We have reorganized the sections in Chapter 3, moving the "Massively Parallel Computing Environment for MVA" section to the beginning of the chapter (renumbered as Section 3.1) to provide a more logical flow of information.
2. We have added an overview paragraph at the beginning of Chapter 3 that outlines the structure and content of the chapter, clarifying the connections between the various sections and topics.
3. We have reviewed the English language throughout the manuscript and made minor adjustments to improve clarity and readability.

We believe that these changes have significantly improved the overall structure and readability of the manuscript, making it easier for readers to follow the logical progression of the research and understand the relationships between the different topics discussed. If there are any specific areas or sections that you feel still require further attention or improvement, please let us know, and we will be happy to address them accordingly.

*Reviewer #2: This present paper talks about the parallelization and simulation of MVA to BCMP, the traditionally used and a representative model of a closed queueing network. This work is interesting, however it has few lacks that need to be fixed.*

1. *Comment: The introduction lacks of references.(from lines 53 to 60)*

Response: Thank you for pointing out the lack of references in the introduction. We have addressed this issue by incorporating relevant citations from the additional literature you provided. Specifically:

1. We have added reference [10] to support our discussion on the importance of social distancing in the context of COVID-19.
2. Reference [11] has been included to highlight the application of queueing theory in analyzing transportation systems during the pandemic.
3. Citations [12] and [13] have been added to support our discussion on the advancements in computing power and parallel processing for queueing theory applications.
4. References [15], [16], and [17] have been incorporated to provide more context on the analysis of multiclass queueing networks and their performance evaluation.

These additions strengthen the academic foundation of our introduction and provide readers with valuable resources for further exploration of the topics discussed. The revised introduction now offers a more comprehensive and well-supported overview of the research context and objectives.

(add to introduction)

10.	D.K. Chu, E.A. Akl, S. Duda, K. Solo, S. Yaacoub, H.J. Schünemann, et al., Physical distancing, face masks, and eye protection to prevent person-to-person transmission of SARS-CoV-2 and COVID-19: a systematic review and meta-analysis, The Lancet 395 (2020) 1973–1987.

11.	R. Jiang, S. Shen, Y. Zhang, Analyzing the impact of COVID-19 on transportation systems using queueing theory, Transportation Research Part A: Policy and Practice 145 (2021) 244-262.

12.	Awan, I., Ahmad, B., & Ahmad, S. (2007). Performance analysis of networks of queues under active queue management scheme. Simulation Modelling Practice and Theory, 15(4), 416-425.

13.	Mesheryakov, R., Moiseev, A., Demin, A., Dorofeev, V., & Sorokin, V. (2016). Using Parallel Computing in Queueing Network Simulation. In Key Engineering Materials (Vol. 685, pp. 943–947). Trans Tech Publications, Ltd. https://doi.org/10.4028/www.scientific.net/kem.685.943

15.	S. Lagershausen, Performance analysis of closed queueing networks, Lecture Notes in Economics and Mathematical Systems, Springer, 2013.

16.	K.M. Rege, Multiclass queueing models for performance analysis of computer systems, Sadhana 15 (1990) 355-363.

17.	D. Bertsimas, D. Gamarnik, J.N. Tsitsiklis, Performance analysis of multiclass queueing networks, SIGMETRICS Performance Evaluation Review 27 (1999) 11-14.

2. *Comment: The font size in table 1 need to adjusted in such away that the table can be readable easily.*

Response: Thank you for your feedback regarding the readability of Table 1. In response to your comment, we have adjusted the font size of Table 1 to enhance its readability. The revised table has been embedded within the main text for your review.

We believe that these adjustments will improve the clarity and overall presentation of the data.

3. *Comment: The ralated work section, is not up to date, please update it. The last reference you are using is since 2019 and there have been few works since then related to the closed BCMP queueing network.*

Response: Thank you for your feedback on the Related Work section and Introduction. We have made substantial improvements to address your concerns:

1. Incorporation of recent research: We have added information on recent studies focusing on large-scale closed queueing networks, machine learning integration, and approximation methods (Patel and Bhathawala [19], Li and Fang [20], Osakwe et al. [21], Sato and Yokota [22], Chen and Yuan [23]).
2. COVID-19 related research: We have emphasized the relevance to current social conditions by including studies on social distancing during the pandemic (Chu et al. [10]) and its impact on transportation systems (Jiang et al. [11]).
3. Advancements in computational methods: We have added recent research on computational methods for queueing networks, including parallel computing (Mesheryakov et al. [13]) and active queue management (Awan et al. [12]).
4. Analysis of closed queueing networks: We have strengthened the theoretical foundation for performance analysis of closed queueing networks by incorporating the works of Lagershausen [15], Rege [16], and Bertsimas et al. [17].
5. Expanded scope: The revised sections now cover a broader range of topics, including non-exponential service times, parallel simulation approaches, deep reinforcement learning applications, and approximate analysis techniques for load-dependent service times.
6. Relevance to current trends: We have highlighted the growing integration of queueing theory with machine learning and its application to modern systems such as smart factories.
7. Consistency in referencing: We have updated the reference numbers throughout the sections to maintain consistency with the newly added literature.

These changes provide a more comprehensive and up-to-date overview of the field, better contextualizing our research within current trends and developments in closed queueing network analysis and applications. Additionally, they clearly demonstrate the relevance of our work to contemporary challenges, such as the impact of the COVID-19 pandemic and the latest computational methods.

(add to Introduction)

10.	D.K. Chu, E.A. Akl, S. Duda, K. Solo, S. Yaacoub, H.J. Schünemann, et al., Physical distancing, face masks, and eye protection to prevent person-to-person transmission of SARS-CoV-2 and COVID-19: a systematic review and meta-analysis, The Lancet 395 (2020) 1973–1987.

11.	R. Jiang, S. Shen, Y. Zhang, Analyzing the impact of COVID-19 on transportation systems using queueing theory, Transportation Research Part A: Policy and Practice 145 (2021) 244-262.

12.	Awan, I., Ahmad, B., & Ahmad, S. (2007). Performance analysis of networks of queues under active queue management scheme. Simulation Modelling Practice and Theory, 15(4), 416-425.

13.	Mesheryakov, R., Moiseev, A., Demin, A., Dorofeev, V., & Sorokin, V. (2016). Using Parallel Computing in Queueing Network Simulation. In Key Engineering Materials (Vol. 685, pp. 943–947). Trans Tech Publications, Ltd. https://doi.org/10.4028/www.scientific.net/kem.685.943

15.	S. Lagershausen, Performance analysis of closed queueing networks, Lecture Notes in Economics and Mathematical Systems, Springer, 2013.

16.	K.M. Rege, Multiclass queueing models for performance analysis of computer systems, Sadhana 15 (1990) 355-363.

17.	D. Bertsimas, D. Gamarnik, J.N. Tsitsiklis, Performance analysis of multiclass queueing networks, SIGMETRICS Performance Evaluation Review 27 (1999) 11-14.

(add to related work)

19.	R. Patel, P.H. Bhathawala, Performance analysis of closed queueing networks with multiple classes and non-exponential service times, Journal of Industrial and Management Optimization 17 (2021) 1825–1842.

20.	Z. Li, W. Fang, Parallel simulation of large-scale closed queueing networks with multi-class customers, in: 2020 IEEE 22nd International Conference on High Performance Computing and Communications, 2020, pp. 822–829.

21.	I.C. Osakwe, A. Aneiba, S. Misra, Performance optimization of queuing systems using deep reinforcement learning, in: Machine Learning for Predictive Analysis, Springer, Cham, 2021, pp. 25-42.

22.	K. Sato, Y. Yokota, Modeling and performance evaluation of a closed queueing network for a smart factory, in: 2020 IEEE International Conference on Industrial Engineering and Engineering Management (IEEM), 2020, pp. 1165-1169.

23.	X. Chen, D. Yuan, Approximate analysis of closed queueing networks with load-dependent service times, Performance Evaluation 148 (2021) 102180.

4. *Comment: It would also interesting that the authors add a flow chart of the process of their proposed approach on the system solved used.*

Response: Thank you for suggesting the inclusion of flowcharts and a symbol table. We have incorporated two flowcharts to visualize our proposed approaches:

1. Fig. 1 in Section 2.3 illustrates the Parallel Computation Algorithm for Mean Value Analysis (MVA) using Message Passing Interface (MPI).
2. Fig. 2 in Section 2.4 depicts the Event-Driven Parallel Simulation Algorithm for BCMP Queueing Networks.

These flowcharts provide a clear visual representation of our methods, enhancing the understanding of the proposed algorithms. Additionally, we have added detailed explanations of these flowcharts in the text to further clarify the process.

Regarding the symbol table, we have included definitions of all symbols used throughout the paper in the relevant sections. However, if you feel a comprehensive symbol table would be beneficial, we can certainly add one in a future revision.

5. *Comment: Figures 1, 2 and 3 are not placed correctly in the paper.*

Response: Thank you for pointing out the issue with the placement of Figures 1, 2, and 3 in the paper. We have addressed this concern by repositioning the figures to immediately follow their respective references in the text. This change ensures that the figures are presented in a logical order and are easily accessible to the reader when they are mentioned in the manuscript. By placing the figures in close proximity to their corresponding text, we aim to improve the overall readability and flow of the paper, allowing readers to better understand and visualize the concepts being discussed. We appreciate your feedback, as it has helped us enhance the clarity and coherence of our work.

6. *Comment: The presented results are promising, however, for a better fainess of the study, the authors are requested to do a comparison study to state of the art with other approaches (if there exist few) for the same datasets in order to show the performance quality of the proposed algorithm.*

Response: We appreciate the reviewers' comments on the importance of comparing our results with those of other studies in the same area. In this study, we have focused on applying the closed BCMP queueing network to a large-scale real-world model, which distinguishes our work from previous studies.

As shown in Table 1, our study considers a significantly larger number of nodes, customer classes, and people in the network compared to earlier research. This demonstrates the novelty and importance of our work in addressing the challenges of analyzing large-scale systems. We have highlighted this point in the revised Section 1.2 "Contributions of this study" as follows:

"i. For closed BCMP, we used MVA, an exact method for obtaining theoretical solutions, to show the limits computable in a modern computing environment. The performance-evaluation index of the closed BCMP could be obtained adequately for large-scale networks, especially when the number of customer classes was three or less. The scale of the systems considered in this study, in terms of the number of nodes, customer classes, and people in the network, is significantly larger than those in previous studies, as shown in Table 1. This demonstrates the novelty and importance of our work in tackling the challenges of large-scale system analysis."

Moreover, we have successfully parallelized the Mean Value Analysis (MVA) algorithm, enabling us to calculate theoretical values for large-scale systems with up to three customer classes. This contribution is significant, as it allows for the analysis of large-scale systems that were previously difficult to handle using conventional methods.

Regarding the simulations, we first verified the accuracy of our approach for large-scale systems with three or fewer customer classes. We then applied simulations to systems with a larger number of customer classes, demonstrating that our proposed method can produce results even for these more complex scenarios. This highlights the effectiveness and broad applicability of our approach. We have emphasized this contribution in the revised Section 1.2:

"iii. Using simulations with verified accuracy, we calculated performance metrics for closed BCMP with four or more customer classes. This simulation-based approach serves as an efficient alternative to exact methods for analyzing large-scale closed BCMP queueing networks that were previously intractable."

While we acknowledge the importance of comparing our results with those of other studies, the unique scale and focus of our work make direct comparisons challenging. However, we believe that our study contributes to the advancement of the field by addressing the limitations of existing methods and providing a novel approach for analyzing large-scale closed BCMP queueing networks.

We believe these revisions have significantly improved the quality of our manuscript, and we hope it now meets the standards of your esteemed journal.

Thank you for your time and consideration.

Sincerely,

Shinya Mizuno

Juntendo University, Faculty of Health Data Science, 6-8-1, Hinode, Urayasu City, Chiba
279-0013, Japan
